# Novel gene expression responses in the ovine abomasal mucosa to infection with the gastric nematode *Teladorsagia circumcincta*

**DOI:** 10.1186/1297-9716-42-78

**Published:** 2011-06-17

**Authors:** Pamela A Knight, Susan E Griffith, Alan D Pemberton, Judith M Pate, Lauren Guarneri, Katherine Anderson, Richard T Talbot, Sarah Smith, David Waddington, Mark Fell, Alan L Archibald, Stewart TG Burgess, David W Smith, Hugh RP Miller, Ivan W Morrison

**Affiliations:** 1 The Roslin Institute and R(D)SVS, University of Edinburgh, Roslin, Midlothian, EH25 9RG, Scotland, UK; 2 Moredun Research Institute, Pentlands Science Park, Bush Loan, Penicuik, Midlothian, EH26 0PZ, Scotland, UK

## Abstract

Infection of sheep with the gastric nematode *Teladorsagia circumcincta *results in distinct Th2-type changes in the mucosa, including mucous neck cell and mast cell hyperplasia, eosinophilia, recruitment of IgA/IgE producing cells and neutrophils, altered T-cell subsets and mucosal hypertrophy. To address the protective mechanisms generated in animals on previous exposure to this parasite, gene expression profiling was carried out using samples of abomasal mucosa collected pre- and post- challenge from animals of differing immune status, using an experimental model of *T. circumcincta *infection. Recently developed ovine cDNA arrays were used to compare the abomasal responses of sheep immunised by trickle infection with worm-naïve sheep, following a single oral challenge of 50 000 *T. circumcincta *L3. Key changes were validated using qRT-PCR techniques. Immune animals demonstrated highly significant increases in levels of transcripts normally associated with cytotoxicity such as granulysin and granzymes A, B and H, as well as mucous-cell derived transcripts, predominantly calcium-activated chloride channel 1 (*CLCA1*). Challenge infection also induced up-regulation of transcripts potentially involved in initiating or modulating the immune response, such as heat shock proteins, complement factors and the chemokine *CCL2*. In contrast, there was marked infection-associated down-regulation of gene expression of members of the gastric lysozyme family. The changes in gene expression levels described here may reflect roles in direct anti-parasitic effects, immuno-modulation or tissue repair. (Funding; DEFRA/SHEFC (VT0102) and the BBSRC (BB/E01867X/1)).

## Introduction

Parasitic gastroenteritis (PGE), caused by trichostrongylid nematodes, is the most commonly diagnosed systemic disease of sheep in the U.K. The principal causative nematode is the abomasal parasite *Teladorsagia circumcincta*. Control currently depends on the use of anthelmintics, but is failing due to the rapid emergence of drug resistance in the target nematodes [1]. Immunity builds up slowly on repeated exposure to the parasite, indicating vaccination could be a feasible alternative, but vaccine development is hampered by a lack of knowledge of the host-parasite interaction to infective larvae. This immunity can be replicated experimentally by giving animals a low level trickle infection over several weeks, which results in a significant level of protective immunity to *T. circumcincta *challenge, measurable by reduced worm burdens, stunting of the worms and increased levels of larval arrest [2-4]. Protective immunity includes both cellular and humoral components; previously exposed animals undergo a local blast cell response in the draining lymphatics which can convey protection to genetically identical naïve recipients [2,5], while local IgA/IgE responses have been associated with certain protective responses such as stunted growth and reduced fecundity of the worms [4,6,7]. *T. circumcincta *challenge in previously immunised sheep elicits local predominantly Th2 cytokine expression, compared to a more Th1-bias in naïve animals [8,9]. This response is accompanied by distinct Th2-type changes in the mucosa, such as mucous neck cell and mast cell hyperplasia, eosinophilia, recruitment of IgA/IgE producing cells and neutrophils, altered T-cell subsets and mucosal hypertrophy [10-14]. However, the molecular changes involved, and the relative contributions of these factors to both control of infection and the clinical symptoms of disease, are still poorly understood. The host immune responses may act concordantly to generate an unfavourable micro-environment [15], which could involve generation of specific antibodies to reduce worm fecundity or feeding [6,7], or promote rapid expulsion [16]. To identify the molecular changes generated in the abomasal mucosa in animals after previous exposure to this parasite, gene expression profiling was carried out using ovine cDNA microarrays on samples of abomasal mucosa collected pre- and post-challenge from animals of differing immune status. The significance of the key changes observed is discussed.

## Materials and Methods

All experimental research described in this manuscript was carried out in accordance with Moredun Research Institute, Roslin Institute and R(D)SVS guidelines. All experimental protocols were approved by the Moredun Research Institute Experiments and Ethical Review Committee and authorised under the UK Animals (Scientific Procedures) Act 1986.

### Infections and sample collection

A series of experimental trials were set up to compare the immune responses of "previously infected" yearling sheep immunised by an eight-week trickle infection (referred to subsequently as "immune" throughout this manuscript), with worm-free naïve yearling sheep, at different timepoints post-challenge with a single dose of 50 000 *T. circumcincta *L3. The details of these infection trials are summarised in Table 1. All sheep were housed under worm-free conditions. Previously described work has established that there were significant differences in anti-parasite responses between the "naïve" and "immune" groups used in this study [4,11]. Samples of host material collected post-mortem included abomasal fold (anterior to the fundic region) for RNA extraction [17]. RNA was extracted as described previously [17] and assessed for quality and quantity using an Agilent 2100 Bioanalyzer (Agilent Technologies UK Ltd, Edinburgh, U.K.) according to ARK-Genomics standard protocols [18].

**Table 1:** Design of *T. circumcincta *infection trials in yearling sheep

Trial	Group	Treatment		**Days post-challenge**^**a**^**; no. of sheep**				
		**Trickle infn**^**b**^	**clearance**^**c**^	**0**^**d**^	**2**	**5**	**10**	**21**

Expt. 1	Naïve (Nv)	-	+	6	-	6	6	6
	Immune (Im)^e^	+	+	-	-	6	6	-

Expt. 2	Naïve (Nv)	-	+	6	6	-	-	-
	Immune (Im)^e^	+	+	6	6	-	-	-

The experimental model of infection used in this study is summarised. Details are given in the footnotes. Two experimental trials were carried out; Expt.1; addressing differences between immune ("previously infected") and naïve sheep at days 5 and 10 post challenge, and Expt.2, addressing differences between immune and naïve and sheep without challenge (day 0) and early post-challenge (day 2).

^a^1 × 50 000 T. *circumcincta *L3 on Day 0

^b^2000 T. *circumcincta *L3 3 × per wk for eight weeks

^c^Levamisole (7.5 mg/kg) 7 days before challenge

^d^Day 0 = unchallenged

^e^previously infected by trickle infection

### Hybridisations and statistical analysis

Ovine cDNA microarrays (ARK-Genomics *O. aries *12 K v1.0) were generated at ARK-Genomics using PCR-amplified products from individual cDNA clones predominantly from the KN511 library; a normalised cDNA library generated from gastrointestinal tract and lymphoid tissues of worm-free and *T. circumcincta *infected sheep, supplemented with an existing sheep/brain library [19]. Expressed Sequence Tags (ESTs) for each cDNA clone have been deposited in the EMBL/Genbank public DNA sequence databases. The array was annotated on the basis of homologous bovine sequences using Unigene, DfCI Gene Index, Ensembl cDNA library and the International Protein Index.

In order to characterise responses at day 5 (Expt.1) and day 0/day 2 (Expt. 2) post-challenge (Table 1), RNA samples were fluorescently labelled and competitively hybridised to the ovine cDNA microarrays using ARK-Genomics standard protocols [18]. Samples were paired in a dye-balanced arrangement as indicated in Table 2. The groups of samples being paired will be termed throughout this manuscript as Nvd5/Nvd0 (i.e. naive day 5 post-challenge vs. day 0 (unchallenged)), Imd5/Nvd5 (i.e. immune vs. naive day 5 post-challenge), Imd0/Nvd0 (i.e. immune vs. naive day 0 (unchallenged)) and Nvd2/Nvd0 or Imd2/Imd0 (i.e. naive or immune day 2 post-challenge vs. day 0 (unchallenged)), and as shown in Table 2. Scanning and data capture using Bluefuse feature extraction software (BlueGnome Limited, Cambridge, United Kingdom) was undertaken according to ARK-Genomics standard protocols [18]. Log_2 _(intensity ratios) were subjected to spatial and intensity dependent normalisations to remove technical bias by spatial row and column averaging [20] and M-A lowess correction [21]. Means of normalized gene log_2 _(intensity ratios) were compared for treatment effects and treatment-by-dye interactions by ANOVA followed by *t*-tests, using modifications of the Limma package [22]. Statistical analysis to generate gene lists was based on each sample coming from a different sheep, and fitting the two (Expt. 1) or three (Expt. 2) dye-balanced arrangements with a common error variance. *T*-tests were modified by the Limma eBayes correction [23]. Genes with Benjamini & Hochberg false discovery rate ≤ 0.05 [24] were considered to show significantly different expression levels. Normalisations and analyses were weighted by the Bluefuse "confidence" measure [25]. Subsets of the data from Expt. 2; genes whose expression levels were significantly changed (FDR ≤ 0.05) ≥ 1.5 fold from the Imd0/Nvd0 and Imd2/Imd0 gene lists (Table 1)); were analyzed through the use of Ingenuity Pathways Analysis (Ingenuity^® ^Systems (Redwood City, CA, U.S.A) [26]) by inputting human gene symbols (HUGO) for their putative ovine orthologues.

**Table 2:** Hybridisation design

Experiment	Hybridisations (d = days post-challenge)		
**Expt. 1**	Nvd0/Nvd5	Nvd5/Imd5	
**Day 5 **post-challenge	(*n *= 6)	(*n *= 6)	

**Expt. 2**	Nvd2/Nvd0	Imd0/Nvd0	Imd2/Imd0
**Day 2 **post-challenge	(*n *= 5^a^)	(*n *= 5^a^)	(*n *= 5^a^)

Samples in both cDNA hybridisation experiments were paired as indicated with a dye-balanced arrangement (three cy3/cy5 & three cy5/cy3 hybridisations). Nv = "naïve" yearlings, worm naïve prior to challenge; Im = "immune" yearlings, previously infected by trickle infection prior to challenge (see Table 1). Common samples linked the sets of paired hybridisations (Expt 1: Nvd5 samples, Expt. 2: Nvd0 & Imd0 samples). An indirect comparison between the results from all three hybridisations gave the (Nvd2/Nvd0)/(Imd2/Imd0) gene list. ^a^One chip failed hybridisation QC in Expt 2 and so was removed from the analysis, along with chips linked to it.

### RT-PCR and multiplex qRT-PCR analysis

Subsequent validation assays for selected groups of genes whose expression levels were significantly changed (FDR ≤ 0.05) ≥ 2 fold were carried out initially by semi-quantitative RT-PCR and sequence analysis of products to confirm identity. Methods for reverse transcription, polymerase chain reaction and purification of PCR products have been described previously [17,27]. Gene-specific primers were designed from the ovine sequences (ESTs) for the relevant KN511 cDNA clones, or previously published sequences, using the Primer 3 program; [28]. Primers and PCR conditions for RT-PCR are shown in Additional file 1: Table S1. Three "housekeeping" genes found to show no significant change in any of the array analyses (*ATPase*, *RW1 *and *TM57*; Table 3) were used as positive controls. Sequence analysis of PCR products was carried out using multiple alignment and sequence similarity search programs available on [29,30] and [31].

**Table 3:** Details of gene sequences, and likely function, selected for further analysis

Functional Category	Gene Name	Clone/probe ID on KN511 cDNA array	**EMBL acc. no**.	Assay
Cytotoxicity	GNLY	C0009264H11.P1KAM13F	FE028690	W1
	CatC	sh1-aoo11_l0.s2	DY479012	W2
	GZMA	KN511_9486f02	FE037399	W2
	GZMB	KN511_9253n09	FE021442	W1
	GZMH	KN511_9260m05	FE026184	W2
Mucus composition	CLCA1	020502OAPP1012080HT OAPP	EE748540	W1
	ITLN1	-^a^	AM087961	W2
	ITLN2	KN511_9264a24.p1kaM13F	EF521881	W2
	ITLN3	-^a^	AM888394	W2
Heat shock response	HSPA8	KN511_9479o03	DY491193	W1
	HSPCA	KN511_9253c04	FE020983	W2
	ST1	KN511_9486e08	FE037366	W1
Proinflammatory response	PLA2G2A	KN511_9482g22	FE034658	W1
	CF1	KN511_9257m10	FE024140	W1
	CCL2	KN511_9477e07	FE031007	W1
Tissue remodelling	MMP13	KN511_9487m11.p1kM13F	AY091604	W1
	MMP23	sh1-bjd04_l0.s2	DY483383	W2
	CST3	KN511_9476g20	FE030675	W1
Digestion	LZM1A	KN511_9487n22.p1kM13F^b^	M32492	W1
	LZM4A	KN511_9487n22.p1kM13F^b^	M32497	W1
Unknown	MALAT	KN511_9484l14	FE036272	W1
"Housekeeping" genes	ATPase	KN511_9488b23.p1kM13F	X02813	W1/W2
	RW1	KN511_9261h17	FE026635	W1/W2
	TM57	KN511_9488i21	FE030125	W1/W2

The EMBL accession no. for the sequences used to design multiplex competitive qRT-PCR assays (Sequenom^® ^MassARRAY^® ^System) is given, together with the representative probe on the ovine cDNA array. The two multiplex competitive qRT-PCR assays were designated W1 and W2 as indicated.

^a^Only *ITLN2 *was represented on the arrays.

^b^These cDNA probes would not be able to distinguish between *LZM1A*, *3A *and *4A*.

In order to provide quantitative data on gene expression for the transcripts investigated above, two multiplex competitive qRT-PCR assays were designed from published sequences as summarised in Table 3; which included a total of 21 study genes and 3 "housekeeping" genes. Multiplex competitive qRT-PCR assays were designed by Sequenom Inc. (San Diego, CA, USA) using QGE assay designer software, version 3.4 (Sequenom^® ^MassARRAY^® ^System; [32]. These assays were used to compare levels of transcripts for each gene in naïve sheep with immune sheep at days 0, 2, 5 and 10 and 21 post-challenge, by running the multiplex qRT-PCR assays with cDNA from all 60 RNA samples summarised in Table 1. The assays and data collection were carried out using Sequenom standard protocols for quantitative gene expression [33]. Significant differences were identified using the Mann-Whitney U-test for non-parametric data.

## Results

### Results of microarray analysis

**Figure 1:**
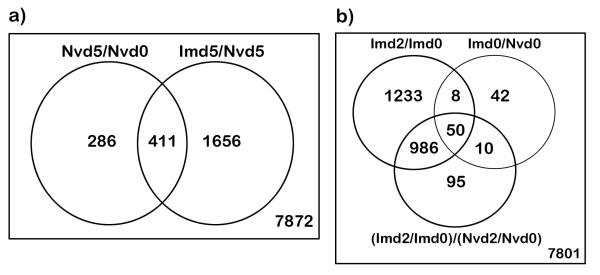
**Venn diagrams to illustrate total numbers of genes whose expression levels were significantly altered (FDR ≤ 0.05) detected in each hybridisation experiment; 1a) Expt.1; day 5 post-challenge 1b) Expt. 2; day 2 post-challenge**. Nv = "naïve" yearlings, worm naïve prior to challenge; Im = "immune" yearlings, previously infected by trickle infection prior to challenge; d0, d2, d5 = days post-challenge; (details in Table 1). The figures in the circled regions correspond to the number of genes with significantly altered (FDR ≤ 0.05) expression levels in each comparison (details given in Table 2) while the figures in the overlapping regions correspond to the number of genes whose expression levels were significantly altered (FDR ≤ 0.05) in two or more comparisons; eg. 8 genes showed significantly altered expression levels in both the Imd2/Imd0 and Imd0/Nvd0 comparisons. The figures outside the circled regions correspond to the numbers of genes on the array with no significant change in expression level in any of the comparisons.

**Figure 2:**
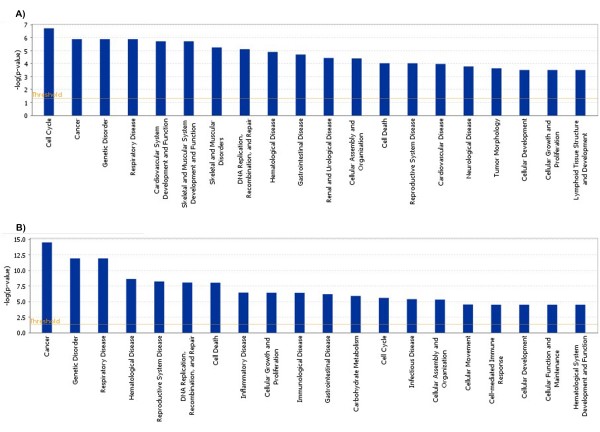
**The top 20 most significant biological functions identified from the Imd0/Nvd0 and Imd2/Imd0 datasets from Expt. 2, using Ingenuity Pathways Analysis software (Ingenuity^® ^Systems**, [26]**)**. The Functional Analysis identified the biological functions and/or diseases that were most significant to the data set. Right-tailed Fisher's exact test was used to calculate a *P*-value determining the probability that each biological function and/or disease assigned to that data set is due to chance alone.

The numbers of transcripts that exhibited significant changes in expression levels (Benjamini & Hochberg FDR ≤0.05) in Expts. 1 and 2 are summarised in the Venn diagrams in Figure 1, and in Additional file 1 Table S2. In both experiments, the highest numbers of transcripts showing significantly altered expression levels (approx > 2K) were associated with the challenged immune group (day 2 or day 5) compared to day 0 of the same group, or compared to naive animals at the same timepoint post-challenge (Figure 1a and 1b). There were no significantly differentially expressed genes in the Nvd2/Nvd0 comparison from Expt. 2. Any transcripts showing significant (FDR ≤ 0.05) treatment-by-dye interactions (Additional file 1: Table S2; Expt. 2) were removed from the gene lists. This precautionary measure excluded pairs of treatment estimates (from cy5-cy3 or cy3-cy5 dye orientations) with conflicting signs and some pairs of treatment estimates with the largest proportionate differences in magnitude (graphs not presented). The complete gene lists of significantly differentially expressed genes, with significant treatment-by-dye interactions excluded, are available under Array Express [34] or by contacting the authors. Data from Expt. 2 were analysed through the use of Ingenuity Pathways Analysis (Ingenuity^® ^Systems, [26]). Data were analysed from two datasets of genes whose expression levels were significantly changed (FDR ≤ 0.05) ≥ 1.5 fold in Expt. 2 (see Table 2): Imd0/Nv d0; to highlight changes induced by trickle infection immunisation, and Imd2/Imd0; to highlight early (<48 h) changes induced following challenge of immunised animals. For each differentially expressed ovine transcript of interest the gene identifier (HUGO) for the putative orthologous human gene and the transcript's expression value were uploaded into the application. The output of the top 20 most significant (Fisher's exact test) biological functions are shown in Figure 2. Significant networks (*P *≤ 10^-10^; Fischer's exact test) identified from the Imd0/Nvd0 and Imd2/Imd0 datasets from Expt. 2 are shown in Additional file 2 Table S3. Pathways were identified from the Ingenuity Pathways Analysis (Ingenuity^® ^Systems, [26]) library of canonical pathways that were most significant to the data set. The top 20 most significant canonical pathways from the Imd0/Nvd0 and Imd2/Imd0 datasets from Expt. 2 are shown in Additional file 2 Table S4.

The biological function associated with the most significant changes (Figure 2) in the Imd0/Nvd0 comparison was "Cell Cycle", which also features as the most significant network (Additional file 2 Table S3) probably reflecting the increased cellular turnover occurring in the immune versus naïve abomasum. "Cancer" was the most significant function from the Imd2/Imd0 comparison, which is likely to relate to increased transcription factor activity following challenge of the immunised sheep. It is notable that the functions "Inflammatory Disease", Immunological Disease", "Infectious Disease" and "Cell-mediated Immune Response" were identified in the Imd2/Imd0 comparison only, which is indicative of increased innate and adaptive immune responses within 48 h following challenge. The most significant network from the Imd2/Imd0 comparison is "Hematological Disease, Small Molecule Biochemistry, Cellular Compromise" (Additional file 2 Table S3); further pathway analysis of this subset (data not shown) showed the most significant pathways within this network were the "Complement System", "Granzyme B signalling" (i.e. Granzyme B pathway), "Huntington's Disease signalling" and "NRF2-mediated oxidative stress response"; the latter two pathways predominantly involve heat shock proteins. It is notable that NFkB signalling predominated in many of the pathways within this network. The 20 most significant pathways from the whole Imd0/Nvd0 and Imd2/Imd0 datasets are shown in Additional file 2 Table S4.

**Figure 3:**
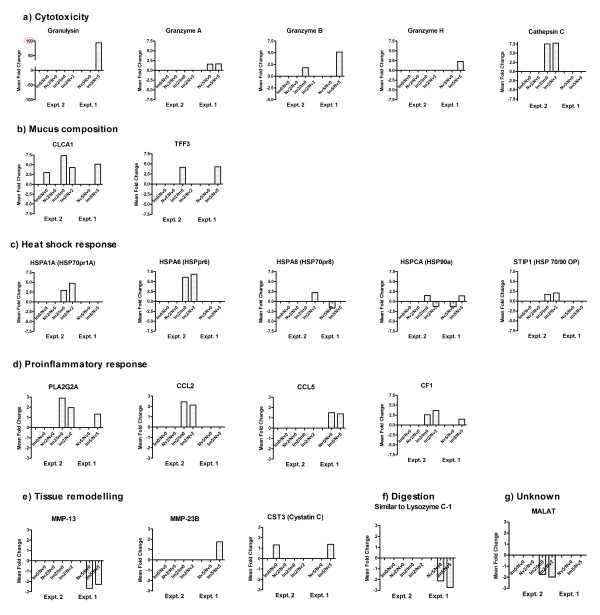
**Key changes detected in Expt. 1 (day 5 post-challenge) and Expt. 2 (day 2 post-challenge)**. Summary of mean infection-associated fold changes in the expression levels of key groups of transcripts detected from the microarray analyses. Transcripts are grouped as follows according to associated function/cell type; a) cytotoxicity, b) mucus composition, c) heat shock response, d) proinflammatory response, e) tissue remodelling (matrix metalloproteinases and inhibitors), f) nutritional (lysozyme family), g) unknown (*MALAT*). The groups being compared are indicated on the y-axis.

**Figure 4:**
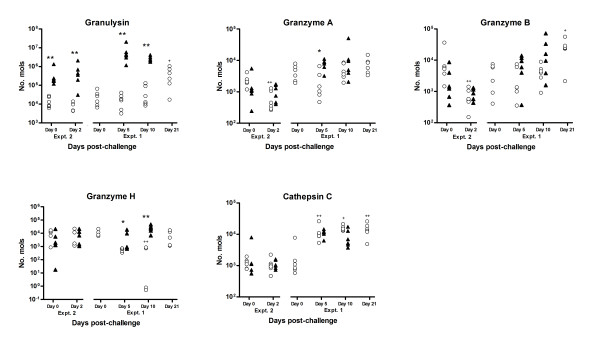
**Results of competitive multiplex qRT-PCR analysis to investigate temporal changes in the expression levels of transcripts normally associated with cells exhibiting cytotoxicity; granulysin, granzymes A, B and H, and cathepsin C, as indicated, throughout the experiment trials summarised in Table 1**. Open circles represent data from naïve sheep; closed triangles represent data from immune ("previously infected") sheep. Significant difference between Nv and Im at same timepoint; *P ≤ 0.05, **P ≤ 0.005, ***P ≤ 0.005; Significant difference between timepoint and day 0 of same group (where available); ^+^*P *≤ 0.05, ^++^*P *≤ 0.005, ^+++^*P *≤ 0.005 (Mann-Whitney U-test for non-parametric data). NB.- data for granzyme H- model fitting failed in some samples, so *n *= 5 in some groups as indicated.

**Figure 5:**
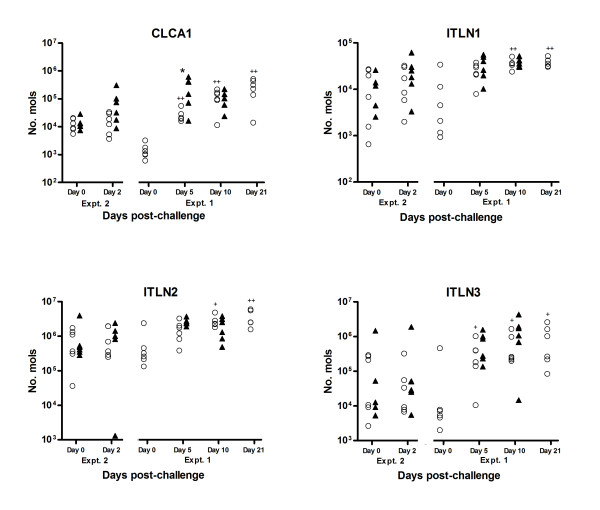
**Results of competitive multiplex qRT-PCR analysis to investigate temporal changes in the expression levels of mucous-cell associated transcripts; *CLCA1 *and *ITLN*s 1-3, as indicated, throughout the experiment trials summarised in Table 1**. Open circles represent data from naïve sheep; closed triangles represent data from immune ("previously infected") sheep. Significant difference between Nv and Im at same timepoint; *P ≤ 0.05, **P ≤ 0.005, ***P ≤ 0.005; Significant difference between timepoint and day 0 of same group (where available); ^+^*P *≤ 0.05, ^++^*P *≤ 0.005, ^+++^*P *≤ 0.005 (Mann-Whitney U-test for non-parametric data).

**Figure 6:**
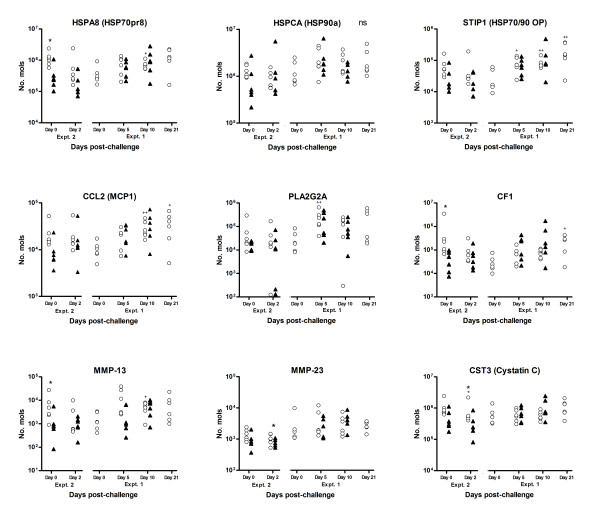
**Results of competitive multiplex qRT-PCR analysis to investigate temporal changes in the expression levels of a number of transcripts associated with tissue remodelling or inflammatory responses throughout the experiment trials summarised in Table 1**. Data is shown for heat shock proteins *HSPA8, HSPCA *and *STIP1*; transcripts associated with pro-inflammatory responses *CCL2*, *PLA2G2A *and *CF1*, and matrix metalloproteinases and inhibitors *MMP-13*, *MMP-23 *and cystatin C, as indicated. Open circles represent data from naïve sheep; closed triangles represent data from immune ("previously infected") sheep. Significant difference between Nv and Im at same timepoint; *P ≤ 0.05, **P ≤ 0.005, ***P ≤ 0.005; Significant difference between timepoint and day 0 of same group (where available); ^+^*P *≤ 0.05, ^++^*P *≤ 0.005, ^+++^*P *≤ 0.005 (Mann-Whitney U-test for non-parametric data).

**Figure 7:**
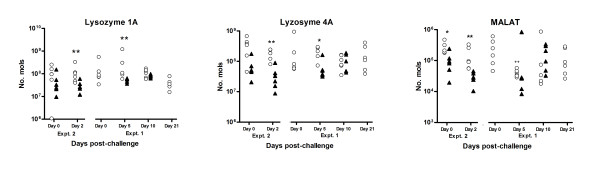
**Results of competitive multiplex qRT-PCR analysis to investigate temporal changes in the expression levels of transcripts for lysozymes 1A and 4A, and *MALAT*, as indicated, throughout the experiment trials summarised in Table 1**. Open circles represent data from naïve sheep; closed triangles represent data from immune ("previously infected") sheep. Significant difference between Nv and Im at same timepoint; *P ≤ 0.05, **P ≤ 0.005, ***P ≤ 0.005; Significant difference between timepoint and day 0 of same group (where available); ^+^*P *≤ 0.05, ^++^*P *≤ 0.005, ^+++^*P *≤ 0.005 (Mann-Whitney U-test for non-parametric data).

The 20 most significantly (FDR ≤ 0.05) up- and down- regulated transcripts from both experiments are shown in Tables 4 (up-regulated transcripts) and 5 (down-regulated transcripts). Fold changes of a selection of these transcripts, grouped under possible function, are illustrated diagrammatically in Figure 3. Transcripts of particular biological interest, listed in Table 3, were selected for further investigation, initially by semi-quantitative RT-PCR (Additional file 1 Table S1) and sequence analysis to validate expression in the abomasum and sequence identity (data not shown) and subsequently, by multiplex competitive qRT-PCR analyses (Table 3). The aims of these assays were to validate the key findings from the array analysis (Figure 3), and to examine temporal changes in response to challenge infection in both the naïve and immune groups, using samples collected at post-mortem on days 0-21 post-challenge as summarised in Table 1. The results of the multiplex qRT-PCR analyses are shown in Figures 4, 5, 6 and 7. The results are discussed below in the context of transcripts specific to particular cell types and/or likely to share a similar function.

**Table 4:** The 20 most up-regulated transcripts detected in Expts. 1 and 2 (FDR ≤ 0.05)

Sequence.ID	TIGR_TC	TC. description	Gene symbol	Fold Change
**EXPT 1. Comparison = Nvd5/Nvd0**				
KN511_9480n02.p1kaM13F	TC385850	---	---	5.44
C0009213B13	---	---	---	1.81
KN511_9261j08.p1kaM13F	---	---	---	1.77
KN511_9255d18.p1kaM13F	TC307335	laminin B1	LAMB1	1.64
KN511_9486f02.p1kM13F	TC305120	UP|GRAA_BOVIN (Q7YRZ7) Granzyme A precursor, complete	gzmA	1.59
KN511_9488i01.p1kM13F	TC311345	homologue to UP|Q96IL1_HUMAN (Q96IL1) DIAPH1 protein (Fragment), partial (43%)	LOC786565	1.55
KN511_9483h09.p1kM13F	TC302202	UP|Q3T119_BOVIN (Q3T119) FCGRT protein, complete	FCGRT	1.53
CN822687	TC381381	similar to emb|X79547.1|MIECCOMP Equus caballus mitochondrial DNA complete sequence, partial (4%)	---	1.52
KN511_9257n17.p1kaM13F	---	---	---	1.52
KN511_9479d15.p1kaM13F	TC361081	similar to UP|O97916_BOVIN (O97916) Reverse transcriptase-like, partial (43%)	---	1.51
KN511_9486m17.p1kM13F	TC303503	UP|C1QA_BOVIN (Q5E9E3) Complement C1q subcomponent subunit A precursor, complete	C1QA	1.50
KN511_9257l13.p1kaM13F	---	---	---	1.50
KN511_9479o01.p1kaM13F	TC355858	GB|AAI02065.1|74353831|BC102064 CCL5 protein {Bos taurus} (exp = -1; wgp = 0; cg = 0), complete	CCL5	1.50
CN823601	TC342786	---	---	1.50
KN511_9486d22.p1kM13F	TC337098	UP|Q3SZV9_BOVIN (Q3SZV9) Heat shock 10kDa protein 1 (Chaperonin 10), complete	HSPE1	1.50
KN511_9477j24.p1kaM13F	TC301808	UP|Q3ZC91_BOVIN (Q3ZC91) Sphingomyelin phosphodiesterase, acid-like 3A, complete	LOC781963	1.48
KN511_9485e22.p1kM13F	---	---	---	1.48
KN511_9253n18.p1kaM13F	TC317864	UP|Q3KZ51_SCHJA (Q3KZ51) SJCHGC03568 protein (Fragment), partial (6%)	MGC139367	1.48
KN511_9265g17.p1kaM13F	TC319596	homologue to UP|TCEA3_HUMAN (O75764) Transcription elongation factor A protein 3 (Transcription elongation factor S-II protein 3) (Transcription elongation factor TFIIS.h), complete	MGC137536	1.48
KN511_9265b23.p1kaM13F	---	---	---	1.48
**EXPT 1 Comparison = Imd5/Nvd5**				
KN511_9264h11.p1kaM13F	TC331976	homologue to UP|Q864L7_BOVIN (Q864L7) Granulysin/NK-lysin-like protein (Fragment), complete	GNLY	94.0
KN511_9257k12.p1kaM13F	---	---	---	5.91
KN511_9263m23.p1kaM13F	TC306994	similar to UP|Q9TUB5_PIG (Q9TUB5) Epithelial chloride channel protein, partial (95%)	CLCA1	5.15
KN511_9253n09.p1kaM13F	TC328399	similar to UP|Q67BC3_HUMAN (Q67BC3) Endogenous granzyme B, partial (90%)	LOC508646	5.14
KN511_9265g07.p1kaM13F	TC351879	---	LOC614719	4.70
KN511_9260m05.p1kaM13F	---	---	---	4.64
KN511_9260b08.p1kaM13F	TC330838	---	---	4.27
CN823175	---	---	---	3.14
CN823115	TC329726	homologue to GB|CAA44699.1|440|BTIGG1HCX anti-testosterone antibody {Bos taurus} (exp = -1; wgp = 0; cg = 0), partial (70%)	IGHG1	2.72
KN511_9480o15.p1kaM13F	---	---	---	2.66
KN511_9476e05.p1kaM13F	TC330547	similar to UP|NBR1_PONPY (Q5RC94) Next to BRCA1 gene 1 protein (Neighbor of BRCA1 gene 1 protein), partial (23%)	LOC515032	2.64
KN511_9486a03.p1kM13F	TC350940	similar to UP|CAD26_MOUSE (P59862) Cadherin-like protein 26 precursor, partial (11%)	LOC617096	2.47
CO202749	TC366134	similar to UP|Q48MR5_PSE14 (Q48MR5) Uncharacterized protein family UPF0016, partial (58%)	---	2.45
KN511_9486o13.p1kM13F	TC322636	weakly similar to UP|Q9R0M7_MOUSE (Q9R0M7) Aldo-keto reductase AKR1C12, partial (91%)	LOC507734	2.42
CN824147	---	---	---	2.38
CN823337	TC313767	UP|Q2FLD4_METHJ (Q2FLD4) ABC transporter related, partial (6%)	---	2.37
CO203065	TC321552	---	---	2.34
KN511_9487e05.p1kM13F	TC302266	GB|AAC67307.1|1930063|BTU92535 neuronal axonal membrane protein {Bos taurus} (exp = -1; wgp = 0; cg = 0), complete	BASP1	2.30
KN511_9261f23.p1kaM13F	---	---	---	2.29
KN511_9478a01.p1kaM13F	TC347423	similar to UP|GRAH_HUMAN (P20718) Granzyme H precursor (Cytotoxic T-lymphocyte proteinase) (Cathepsin G-like 2) (CTSGL2) (CCP-X) (Cytotoxic serine protease C) (CSP-C), partial (93%)	LOC617313	2.28
KN511_9486l10.p1kM13F	TC315163	similar to GB|AAH12303.1|15126763|BC012303 PDZK1 interacting protein 1 {Homo sapiens} (exp = -1; wgp = 0; cg = 0), complete	PDZK1IP1	2.21
CN824077	---	---	---	2.21
CN824594	TC359921	homologue to UP|Q4K9Y4_PSEF5 (Q4K9Y4) BNR/Asp-box repeat protein, partial (41%)	---	2.14
KN511_9260m24.p1kaM13F	---	---	---	2.13
**EXPT 2 Comparison = Imd0/Nvd0**				
KN511_9484k05.p1kM13F	TC323139	similar to UP|Q4TA31_TETNG (Q4TA31) Chromosome undetermined SCAF7459, whole genome shotgun sequence, partial (10%)	---	3.59
KN511_9263m23.p1kaM13F	TC306994	similar to UP|Q9TUB5_PIG (Q9TUB5) Epithelial chloride channel protein, partial (95%)	CLCA1	2.99
KN511_9484p11.p1kM13F	---	---	---	2.08
KN511_9485e08.p1kM13F	TC312631	---	---	1.61
KN511_9478a06.p1kaM13F	TC312631	---	---	1.49
KN511_9481c23.p1kaM13F	TC314684	---	---	1.46
CN823114	TC301217	homologue to UP|NDRG1_HUMAN (Q92597) Protein NDRG1 (N-myc downstream-regulated gene 1 protein) (Differentiation-related gene 1 protein) (DRG-1) (Reducing agents and tunicamycin-responsive protein) (RTP) (Nickel-specific induction protein Cap43) (Rit42), partial (97%)	NDRG1	1.37
KN511_9257n17.p1kaM13F	---	---	---	1.35
CN823685	TC315004	similar to UP|Q3TT48_MOUSE (Q3TT48) Adult male pituitary gland cDNA, RIKEN full-length enriched library, clone:5330433L19 product:proprotein convertase subtilisin/kexin type 2, full insert sequence. (Fragment), partial (3%)	LOC615685	1.33
KN511_9476g20.p1kaM13F	TC337413	UP|CYTC_BOVIN (P01035) Cystatin C precursor (Colostrum thiol proteinase inhibitor), complete	CST3	1.31
	---	---	---	1.30
KN511_9259b18.p1kaM13F	TC371167	---	---	1.29
KN511_9479a08.p1kaM13F	TC318244	UP|CATA_BOVIN (P00432) Catalase, partial (5%)	MGC128112	1.29
CN823587	---	---	---	1.27
CO202780	TC357183	UP|Q71V68_MOUSE (Q71V68) Ldb1a, complete	LOC526472	1.26
KN511_9262a18.p1kaM13F	TC308953	UP|Q3T013_BOVIN (Q3T013) BCL2/adenovirus E1B 19kDa interacting protein 3-like, partial (84%)	BNIP3L	1.26
CO202648	TC301621	homologue to UP|Q9HB23_HUMAN (Q9HB23) Lysyl-tRNA synthetase, complete	MGC127504	1.25
KN511_9254i12.p1kaM13F	TC381609	homologue to UP|Q963G4_PLAFA (Q963G4) MB2 (Fragment), partial (3%)	LOC508133	1.25
KN511_9261e18.p1kaM13F	---	---	---	1.24
**EXPT 2 Comparison = Imd2/Imd0**				
KN511_9481e19.p1kaM13F	TC318483	---	LRIG1	10.2
KN511_9256h20.p1kaM13F	TC309152	similar to UP|O97916_BOVIN (O97916) Reverse transcriptase-like, partial (19%)	---	10.0
KN511_9257i19.p1kaM13F	TC375873	---	---	9.71
KN511_9481f19.p1kaM13F	TC345827	homologue to GB|CAI11042.1|55859631|AL353665 match: proteins: Q92802 {Homo sapiens} (exp = 0; wgp = 1; cg = 0), partial (35%)	LOC541201	9.17
KN511_9480f18.p1kaM13F	---	---	---	8.94
CN822322	TC309241	nucleolin [Bos taurus]	NCL	8.03
KN511_9483a22.p1kM13F	TC316336	UP|Q3ZCJ8_BOVIN (Q3ZCJ8) Cathepsin C, complete	CTSC	7.52
KN511_9263m23.p1kaM13F	TC306994	similar to UP|Q9TUB5_PIG (Q9TUB5) Epithelial chloride channel protein, partial (95%)	CLCA1	7.33
KN511_9483l13.p1kM13F	TC340529	homologue to UP|Q9BTX0_HUMAN (Q9BTX0) RNA binding motif protein 10, isoform 2, partial (69%)	RBM10	6.73
KN511_9488g12.p1kM13F	TC304325	similar to GB|CAJ18388.1|71059689|CT010180 {Mus musculus} (exp = -1; wgp = 0; cg = 0), complete	MGC127625	6.46
KN511_9263i18.p1kaM13F	TC332274	UP|Q7ZU59_BRARE (Q7ZU59) H2AV protein (Fragment), partial (87%)	H2AFZ	6.45
KN511_9253j02.p1kaM13F	TC331773	homologue to UP|HSP76_PIG (Q04967) Heat shock 70 kDa protein 6 (Heat shock 70 kDa protein B'), complete	HSPA6	6.11
KN511_9487k02.p1kM13F	TC385703	UP|VPS28_MOUSE (Q9D1C8) VPS28 protein homolog (Caspase-activated DNase inhibitor that interacts with ASK1) (CIIA), complete	VPS28	5.97
KN511_9265g07.p1kaM13F	TC351879	---	LOC614719	5.84
KN511_9476g04.p1kaM13F	TC371801	homologue to SP|O60506|HNRPQ_HUMAN Heterogeneous nuclear ribonucleoprotein Q (hnRNP Q) (hnRNP-Q)(Synaptotagmin binding, cytoplasmic RNA interacting protein) (Glycine-and tyrosine-rich RNA binding protein) (GRY-RBP) (NS1-associatedprotein 1). {Homo sapiens} (exp = -1; wgp = -1; cg = -1), partial (51%)	SYNCRIP	5.58
KN511_9263f17.p1kaM13F	TC355165	similar to UP|Q9XSA0_SHEEP (Q9XSA0) Pulmonary surfactant-associated protein B (Fragment), partial (16%)	---	4.64
KN511_9476p04.p1kaM13F	TC329988	UP|Q5E973_BOVIN (Q5E973) Ribosomal protein L18, complete	RPL18	4.47
KN511_9480l18.p1kaM13F	TC364833	---	---	4.38
KN511_9265h18.p1kaM13F	TC303880	homologue to UP|O35328_MOUSE (O35328) Proline-rich protein 9-1 (Fragment), partial (6%)	---	4.18
KN511_9260b08.p1kaM13F	TC330838	--	---	4.15

The experiment and the samples being compared are given as subheadings at the top of each section. Nv = "naïve" yearlings, worm naïve prior to challenge; Im = "immune" yearlings, previously infected by trickle infection prior to challenge, d = days post-challenge. Details of the experimental trials and hybridisation design are given in Tables 1 and 2.

**Table 5:** The 20 most down-regulated transcripts detected in Expts 1 and 2 (FDR ≤ 0.05)

Sequence.ID	TIGR_TC	TC.description	Gene symbol	Fold Change
**EXPT 1 Comparison = d5Nv_d0Nv**				
KN511_9487m11.p1kM13F	TC336019	UP|MMP13_BOVIN (O77656) Collagenase 3 precursor (Matrix metalloproteinase-13) (MMP-13), complete	MMP13	-2.68
KN511_9481c03.p1kaM13F	TC304583	UP|LYSC1_BOVIN (Q06285) Lysozyme C-1 precursor (1,4-beta-N-acetylmuramidase C), complete	LYZ2	-2.37
KN511_9476d13.p1kaM13F	TC304583	UP|LYSC1_BOVIN (Q06285) Lysozyme C-1 precursor (1,4-beta-N-acetylmuramidase C), complete	LYZ2	-2.33
KN511_9485a08.p1kM13F	TC302650	milk lysozyme [Bos taurus]	LYZ1	-2.30
KN511_9485g08.p1kM13F	TC346615	similar to UP|O97916_BOVIN (O97916) Reverse transcriptase-like, partial (32%)	---	-2.23
KN511_9257d12.p1kaM13F	TC322737	similar to UP|Q3VD89_9SPHN (Q3VD89) ABC-2, partial (5%)	---	-2.16
KN511_9257a18.p1kaM13F	TC308928	similar to GB|AAK18773.1|13324523|F272846S31 Fanconi anemia complementation group D2 protein, isoform 2 {Homo sapiens} (exp = -1; wgp = 0; cg = 0), partial (18%)	LOC515845	-2.14
KN511_9262d01.p1kaM13F	---	---	---	-2.12
KN511_9261n23.p1kaM13F	TC362037	homologue to UP|IFRD1_PIG (Q5S1U6) Interferon-related developmental regulator 1, complete	IFRD1	-2.12
KN511_9476d14.p1kaM13F	TC349311	similar to UP|NEK4_HUMAN (P51957) Serine/threonine-protein kinase Nek4 (NimA-related protein kinase 4) (Serine/threonine-protein kinase 2) (Serine/threonine-protein kinase NRK2), partial (33%)	MGC159441	-2.08
KN511_9261o08.p1kaM13F	TC305060	homologue to UP|Q96BA7_HUMAN (Q96BA7) HNRPU protein, partial (93%)	MGC142835	-2.07
KN511_9258d23.p1kaM13F	---	---	---	-2.07
KN511_9487p21.q1kM13R	TC342358	homologue to UP|DC1L2_HUMAN (O43237) Cytoplasmic dynein 1 light intermediate chain 2 (Dynein light intermediate chain 2, cytosolic) (LIC53/55) (LIC-2), complete	LOC519789	-2.05
KN511_9262k18.p1kaM13F	TC375209	similar to UP|ZSWM3_HUMAN (Q96MP5) Zinc finger SWIM domain-containing protein 3, partial (27%)	LOC512244	-2.05
KN511_9485d23.q1kM13R	TC365645	UP|LYSC2_BOVIN (Q06283) Lysozyme C-2 precursor (1,4-beta-N-acetylmuramidase C), complete	LYZ2	-2.04
KN511_9254g13.p1kaM13F	TC319944	---	---	-2.02
KN511_9487h17.p1kM13F	TC327406	UP|Q5DTN6_MOUSE (Q5DTN6) MKIAA4095 protein (Fragment), partial (8%)	LOC614823	-1.96
CO202908	TC360504	UP|Q3MHM4_BOVIN (Q3MHM4) Heat shock 70 kDa protein 8, complete	HSPA8	-1.87
KN511_9482g13.p1kM13F	TC331274	homologue to UP|TENA_PIG (Q29116) Tenascin precursor (TN) (Hexabrachion) (Cytotactin) (Neuronectin) (GMEM) (JI) (Miotendinous antigen) (Glioma-associated-extracellular matrix antigen) (GP 150-225) (Tenascin-C) (TN-C) (P230), partial (10%)	MGC140517	-1.87
KN511_9482f05.p1kM13F	TC377013	---	---	-1.86
**EXPT 1 Comparison = d5Im_d5Nv**				
KN511_9256g17.p1kaM13F	TC314535	homologue to UP|Q94513_DROME (Q94513) Boundary element associated factor (LD44361p) (CG10159-PB, isoform B), partial (5%)	---	-6.51
KN511_9256g17.p1kaM13F	TC314535	homologue to UP|Q94513_DROME (Q94513) Boundary element associated factor (LD44361p) (CG10159-PB, isoform B), partial (5%)	---	-5.97
KN511_9254l23.p1kaM13F	TC354719	similar to UP|TFF2_HUMAN (Q03403) Trefoil factor 2 precursor (Spasmolytic polypeptide) (SP) (Spasmolysin), complete	MGC139191	-5.54
KN511_9257l15.p1kaM13F	TC354719	similar to UP|TFF2_HUMAN (Q03403) Trefoil factor 2 precursor (Spasmolytic polypeptide) (SP) (Spasmolysin), complete	MGC139191	-5.42
KN511_9260b10.p1kaM13F	TC365645	UP|LYSC2_BOVIN (Q06283) Lysozyme C-2 precursor (1,4-beta-N-acetylmuramidase C), complete	LYZ2	-3.92
KN511_9260m19.p1kaM13F	TC304583	UP|LYSC1_BOVIN (Q06285) Lysozyme C-1 precursor (1,4-beta-N-acetylmuramidase C), complete	LYZ2	-3.79
KN511_9483j03.p1kM13F	TC365645	UP|LYSC2_BOVIN (Q06283) Lysozyme C-2 precursor (1,4-beta-N-acetylmuramidase C), complete	LYZ2	-3.52
KN511_9265j02.p1kaM13F	CB220717	---	---	-3.45
KN511_9264e04.p1kaM13F	TC314849	UP|Q6SJV6_BOVIN (Q6SJV6) Foveolin precursor, complete	FOV	-3.33
KN511_9262k17.p1kaM13F	TC304583	UP|LYSC1_BOVIN (Q06285) Lysozyme C-1 precursor (1,4-beta-N-acetylmuramidase C), complete	LYZ2	-3.25
KN511_9476c18.p1kaM13F	TC365645	UP|LYSC2_BOVIN (Q06283) Lysozyme C-2 precursor (1,4-beta-N-acetylmuramidase C), complete	LYZ2	-3.25
KN511_9476d13.p1kaM13F	TC304583	UP|LYSC1_BOVIN (Q06285) Lysozyme C-1 precursor (1,4-beta-N-acetylmuramidase C), complete	LYZ2	-3.17
KN511_9488a15.p1kM13F	TC365645	UP|LYSC2_BOVIN (Q06283) Lysozyme C-2 precursor (1,4-beta-N-acetylmuramidase C), complete	LYZ2	-3.16
KN511_9259k01.p1kaM13F	TC365645	UP|LYSC2_BOVIN (Q06283) Lysozyme C-2 precursor (1,4-beta-N-acetylmuramidase C), complete	LYZ2	-3.14
KN511_9265m20.p1kaM13F	TC304583	UP|LYSC1_BOVIN (Q06285) Lysozyme C-1 precursor (1,4-beta-N-acetylmuramidase C), complete	LYZ2	-3.10
KN511_9263j17.p1kaM13F	TC323852	similar to UP|Q86XP6_HUMAN (Q86XP6) GDDR (Blottin precursor) (TFIZ1 protein precursor), complete	LOC512001	-3.10
KN511_9487e19.p1kM13F	TC365645	UP|LYSC2_BOVIN (Q06283) Lysozyme C-2 precursor (1,4-beta-N-acetylmuramidase C), complete	LYZ2	-3.08
KN511_9485d23.q1kM13R	TC365645	UP|LYSC2_BOVIN (Q06283) Lysozyme C-2 precursor (1,4-beta-N-acetylmuramidase C), complete	LYZ2	-3.05
KN511_9486h17.p1kM13F	TC314849	UP|Q6SJV6_BOVIN (Q6SJV6) Foveolin precursor, complete	FOV	-3.04
KN511_9265h22.p1kaM13F	TC304583	UP|LYSC1_BOVIN (Q06285) Lysozyme C-1 precursor (1,4-beta-N-acetylmuramidase C), complete	LYZ2	-2.99
**EXPT 2 Comparison = Day 0 Im vs Day 0 Nv**				
KN511_9484p13.p1kM13F	TC376600	similar to UP|CENPA_MOUSE (O35216) Centromere protein A (CENP-A) (Centromere autoantigen A), partial (15%)	CENP-A	-1.75
KN511_9479i16.p1kaM13F	---	---	---	-1.75
KN511_9253l11.p1kaM13F	TC303509	homologue to UP|RIR2_HUMAN (P31350) Ribonucleoside-diphosphate reductase M2 subunit (Ribonucleotide reductase small subunit) (Ribonucleotide reductase small chain), partial (88%)	RRM2	-1.73
KN511_9488d17.p1kM13F	TC314207	similar to UP|TOP2A_PIG (O46374) DNA topoisomerase 2-alpha (DNA topoisomerase II, alpha isozyme), partial (17%)	TOP2A	-1.71
KN511_9265l09.p1kaM13F	TC340393	UP|CCNA2_BOVIN (P30274) Cyclin-A2 (Cyclin-A) (Fragment), complete	CCNA2	-1.70
KN511_9488f20.p1kM13F	TC355225	homologue to UP|BIRC5_CANFA (Q8I009) Baculoviral IAP repeat-containing protein 5 (Apoptosis inhibitor survivin), complete	BIRC5	-1.66
KN511_9487m04.p1kM13F	TC355225	homologue to UP|BIRC5_CANFA (Q8I009) Baculoviral IAP repeat-containing protein 5 (Apoptosis inhibitor survivin), complete	BIRC5	-1.65
KN511_9253i15.p1kaM13F	TC340307	UP|CP51A_BOVIN (Q4PJW3) Cytochrome P450 51A1 (CYPLI) (P450LI) (Sterol 14-alpha demethylase) (Lanosterol 14-alpha demethylase) (LDM) (P450-14DM) (P45014DM), complete	CYP51	-1.63
KN511_9265a16.p1kaM13F	TC303827	---	KIF11	-1.58
KN511_9264b09.p1kaM13F	---	---	---	-1.56
KN511_9256j19.p1kaM13F	TC355225	homologue to UP|BIRC5_CANFA (Q8I009) Baculoviral IAP repeat-containing protein 5 (Apoptosis inhibitor survivin), complete	BIRC5	-1.53
KN511_9261d22.p1kaM13F	---	---	---	-1.50
KN511_9481l06.p1kaM13F	TC303509	homologue to UP|RIR2_HUMAN (P31350) Ribonucleoside-diphosphate reductase M2 subunit (Ribonucleotide reductase small subunit) (Ribonucleotide reductase small chain), partial (88%)	LOC508167	-1.49
KN511_9476j14.p1kaM13F	TC333525	homologue to GB|AAH08056.2|39644830|BC008056 PLEK2 protein {Homo sapiens} (exp = -1; wgp = 0; cg = 0), partial (21%)	MGC142714	-1.48
KN511_9482e10.p1kM13F	TC330132	similar to UP|CV106_HUMAN (Q6P0N0) Protein C14orf106 (P243), partial (33%)	LOC507661	-1.47
KN511_9257p16.p1kaM13F	TC340307	UP|CP51A_BOVIN (Q4PJW3) Cytochrome P450 51A1 (CYPLI) (P450LI) (Sterol 14-alpha demethylase) (Lanosterol 14-alpha demethylase) (LDM) (P450-14DM) (P45014DM), complete	CYP51	-1.46
KN511_9256f06.p1kaM13F	TC332145	similar to UP|Q9BDH4_PIG (Q9BDH4) Amphiregulin long form, complete	MGC152310	-1.45
CN824006	TC306385	homologue to UP|Q4S6A0_TETNG (Q4S6A0) Chromosome 9 SCAF14729, whole genome shotgun sequence, partial (59%)	LOC404053	-1.42
KN511_9257i02.p1kaM13F	TC327524	homologue to UP|Q9BW51_HUMAN (Q9BW51) TTK protein kinase, partial (44%)	LOC524925	-1.41
KN511_9261e22.p1kaM13F	TC301804	homologue to UP|IMA2_HUMAN (P52292) Importin alpha-2 subunit (Karyopherin alpha-2 subunit) (SRP1-alpha) (RAG cohort protein 1), complete	KPNA2	-1.41
**EXPT 2 Comparison = Day 2 Im vs Day 0 Im**				
KN511_9479k18.p1kaM13F	CX950602	---	---	-3.53
KN511_9488b02.p1kM13F	DY126632	---	---	-3.24
KN511_9257l15.p1kaM13F	TC354719	similar to UP|TFF2_HUMAN (Q03403) Trefoil factor 2 precursor (Spasmolytic polypeptide) (SP) (Spasmolysin), complete	TFF2	-3.14
KN511_9254l23.p1kaM13F	TC354719	similar to UP|TFF2_HUMAN (Q03403) Trefoil factor 2 precursor (Spasmolytic polypeptide) (SP) (Spasmolysin), complete	TFF2	-3.03
KN511_9264e04.p1kaM13F	TC314849	UP|Q6SJV6_BOVIN (Q6SJV6) Foveolin precursor, complete	FOV	-2.81
KN511_9263j17.p1kaM13F	TC323852	similar to UP|Q86XP6_HUMAN (Q86XP6) GDDR (Blottin precursor) (TFIZ1 protein precursor), complete	GKN2	-2.45
KN511_9486h17.p1kM13F	TC314849	UP|Q6SJV6_BOVIN (Q6SJV6) Foveolin precursor, complete	FOV	-2.09
KN511_9261k22.p1kaM13F	TC327109	similar to UP|O97916_BOVIN (O97916) Reverse transcriptase-like, partial (21%)	---	-1.93
KN511_9486b13.p1kM13F	TC341767	---	---	-1.90
KN511_9484h13.p1kM13F	TC350450	similar to UP|UDB5_MOUSE (P17717) UDP-glucuronosyltransferase 2B5 precursor (UDPGT) (M-1), partial (49%)	UGT2B17	-1.81
KN511_9263c22.p1kaM13F	---	---	---	-1.80
KN511_9264a19.p1kaM13F	---	---	---	-1.77
KN511_9484l14.p1kM13F	TC362885	similar to UP|MALAT_HUMAN (Q9UHZ2) Metastasis-associated lung adenocarcinoma transcript 1, partial (72%)	MALAT1	-1.75
KN511_9480l06.p1kaM13F	TC362885	similar to UP|MALAT_HUMAN (Q9UHZ2) Metastasis-associated lung adenocarcinoma transcript 1, partial (72%)	MALAT1	-1.74
KN511_9481o23.p1kaM13F	TC350450	similar to UP|UDB5_MOUSE (P17717) UDP-glucuronosyltransferase 2B5 precursor (UDPGT) (M-1), partial (49%)	UGT2B17	-1.73
CN824758	---	---	---	-1.72
KN511_9488a17.p1kM13F	TC318925	---	---	-1.71
KN511_9480o03.p1kaM13F	---	---	---	-1.70
KN511_9263o20.p1kaM13F	TC380127	similar to UP|UDB5_MOUSE (P17717) UDP-glucuronosyltransferase 2B5 precursor (UDPGT) (M-1), partial (42%)	UGT2B17	-1.70
KN511_9484i14.p1kM13F	TC362885	similar to UP|MALAT_HUMAN (Q9UHZ2) Metastasis-associated lung adenocarcinoma transcript 1, partial (72%)	MALAT1	-1.67

The experiment and the samples being compared are given as subheadings at the top of each section. Nv = "naïve" yearlings, worm naïve prior to challenge; Im = "immune" yearlings, previously infected by trickle infection prior to challenge, d = days post-challenge. Details of the experimental trials and hybridisation design are given in Tables 1 and 2.

### Transcripts normally associated with cells exhibiting cytotoxicity

Transcripts for granulysin were identified as the most highly up-regulated in immune sheep (+94 fold), compared with naïve at day 5 post-infection (p.i.) (Table 4; Figure 3), and were confirmed to show significantly (FDR ≤ 0.05) highly up-regulated expression in immune animals at all timepoints post-challenge by multiplex qRT-PCR (Figure 4). We also observed a +2-5 fold up-regulation of transcripts for granzymes A, B and H in immune animals on day 5 post-challenge compared with naïve, and a >7 fold up-regulation of cathepsin C transcripts in immune animals on day 2 after challenge compared to naïve or to day 0 (Figure 3a). Results from the multiplex qRT-PCR analysis showed significantly higher numbers of transcripts for granzymes A and H in immune abomasal samples compared to naïve at day 5 post-challenge, with elevated levels persisting at day 10, but expression of granzyme B was highly variable and not significant (Figure 4). Transcripts for cathepsin C were generally 10-fold more abundant in challenged animals at days 5-21 compared to day 0, although there were no significant differences between the immune and naïve groups (Figure 4). The 20 most significant pathways identified from the Imd2/Imd0 comparison included "Crosstalk between Dendritic cells and NK cells", "Granzyme B Signalling" and "Leukocyte Extravasation Signalling", indicative of an increased cell-mediated immune response (Additional file 2 Table S4).

### Mucous-cell associated transcripts

The mucous-cell associated transcript calcium-activated chloride channel 1 (*CLCA1*) was one of the most up-regulated transcripts seen in immune sheep in both experiments. *CLCA1 *was the second most up-regulated transcript in immune versus naïve sheep day 5 post-challenge (Expt. 1) and was also among the most up-regulated transcripts in the immune versus naïve sheep at day 0 and day 2 post-challenge (Expt 2) (Table 4, Figure 3b)). Significant (FDR ≤ 0.05) up-regulation of *CLCA1 *transcripts in immune versus naïve sheep was confirmed in the multiplex qRT-PCR analysis (day 5 post-challenge, Figure 5) and there was a general up-regulation of expression in response to challenge (i.e. compared to day 0) in both groups, significant (*P *≤ 0.01) in naïve animals on days 5-21. We also observed significant up-regulation of transcripts for the mucous cell product trefoil factor 3 (*TFF3*) in immune animals compared to naïve (Table 4; Figure 3b)), while trefoil factor 2 (*TFF2*) was highly down-regulated (Table 5).

While members of the intelectin family, galactose-binding lectins expressed by mucous neck cells [27], did not feature in the lists of significantly up-regulated genes from the microarray analysis (only *ITLN2 *(KN511_9264a24.p1kaM13F) was represented on the array), specific assays for all three of these genes were included in the multiplex qRT-PCR analysis as a positive control, since *ITLN*s 1, 2 and 3 have been shown to be up-regulated in abomasal mucosa in response to *T. circumcincta *[35]. There was a significant up regulation in response to challenge in the naïve group for all 3 intelectins, with earlier up-regulation of *ITLN-3 *than *ITLN-1 *and *-2*, although these assays did not show significant differences between naïve and immune sheep (Figure 5). We also observed infection-associated up-regulation of transcripts for two galectins, which belong to the C-type lectin family; galectin 1 (+1.5 fold), and galectin 4 (+1.6 fold).

### Mast -cell associated transcripts

Significantly (FDR ≤ 0.05) up-regulated gene expression of the high affinity IgE receptor in immune animals was detected on day 2 (+1.5 fold) and day 5 (+1.8 fold) post-challenge, compared to the naïve group at the same timepoints. Up-regulated expression of cathepsin C, another potential mast cell transcript, has already been mentioned in the previous section. *SMCP-1 *and tryptase, which are expressed by abomasal mucosal mast cells during nematode infection [36,37], did not feature on this array.

### Heat shock proteins

Significant up-regulation of a number of transcripts for heat shock proteins was detected in both microarray experiments. Transcripts for the heat shock proteins *HSP1A *(HSP70 protein 1A) and *HSPA6 *(HSP 70 protein 6) feature among some of the most significantly (FDR ≤ 0.05) highly up-regulated transcripts detected in immune sheep, up-regulated (+3-7 fold) by day 2 post-challenge in this group in comparison to naive (Figure 3c) and Table 4). The *STIP1 *(HSP70/90 organising protein; HOP) and *HSPA8 *(HSP70 protein 8) genes also appear to show significantly up-regulated expression in immune animals compared to naïve (+1.5-2 fold) (Figure 3c) and Table 4). Transcripts for *HSPCA *(HSP90a) appear to show significantly (FDR ≤ 0.05) increased levels on day 2 post-challenge in immune animals, although the expression pattern varies across the different comparisons (Figure 3c) and Table 4). The most significant network identified in the Imd2/Imd0 comparison (Network 1;"Cellular Compromise"; Additional file 2 Table S3) links *STIP1 *with a number of heat shock proteins in the *HSP70/90 *family. Multiplex qRT-PCR analysis showed that transcripts for *STIP1 *were significantly (*P *≤ 0.01-0.05) increased day 5-21 post-challenge in the naïve group, with a similar trend in immune sheep, although there were no immune day 0 animals within the same trial (Figure 6). Multiplex qRT-PCR analysis, however, did not support all the findings for *HSPA8 *and *HSPCA*, although there was a significant (*P *≤ 0.05) challenge-associated increase in levels of *HSPA8 *transcripts in the naïve group by day 10 (Figure 6).

### Transcripts associated with pro-inflammatory responses

The microarray analyses demonstrated significant (FDR ≤ 0.05) up-regulation of a number of transcripts associated with eicosanoid metabolism. Transcripts for *PLA2G2A*, which liberates arachidonic acid from phospholipids, were significantly up-regulated in response to challenge (+3 fold), and in immune animals compared to naïve at day 2 and day 5 post-challenge (+1.5-2 fold) (Figure 3d). The multiplex qRT-PCR analysis showed significant (*P *≤ 0.05) up-regulation of *PLA2G2A *transcripts at day 5 post-challenge, but did not show significant differences between the naïve and immune groups (Figure 6). Transcripts for prostaglandin E synthase (*CPGES*), another member of this pathway, appear to be significantly (FDR ≤ 0.05) up-regulated (+1.6 fold) in immune animals by day 2 post-challenge compared to naïve animals. "Arachidonic Acid Metabolism" features among the significant pathways from the Imd0/Nvd0 comparison (Additional file 2 Table S4).

Transcripts for the chemokine *CCL2 *(MCP-1) were significantly up-regulated >2 fold on day 2 post-challenge in immune sheep, compared to naïve or unchallenged sheep (Figure 3d). Multiplex qRT-PCR analysis showed that transcripts for *CCL2 *were significantly (*P *≤ 0.01-0.05) increased day 10-21 post-challenge in the naïve group in Expt. 1, with a similar trend in the immune group, but did not show significant differences between the two groups (Figure 6). Transcripts for the chemokine *CCL5 *(RANTES) were among the most up-regulated in naïve sheep on day 5 post-challenge (+1.5 fold; Figure 3d)), and were up-regulated in immune sheep compared to naive at this timepoint (+1.4 fold).

Gene expression of several members of the complement cascade also appear to be up-regulated in response to *T. circumcincta *challenge. Complement factor 1 (CF1), and other members of the classical C1-complex, were significantly (FDR ≤ 0.05) up-regulated in immune sheep in response to challenge by day 2 (+2-3 fold), but not in naïve animals (Table 4 and Figure 3d)). The "Complement System" was the third most significant pathway identified from the Imd2/Imd0 comparison (Figure 2), and the most significant pathway within the Network 1 subset from this comparison (Additional file 2 Table S3). However, the multiplex qRT-PCR analysis specific for complement factor 1 did not show the same pattern of expression detected in the arrays, with a lot of sheep-to-sheep variation, although there was a clear trend towards up-regulated gene expression in response to challenge in both groups in Expt. 1 (Figure 6).

### Matrix metalloproteinases and protease inhibitors

Transcripts for the matrix metalloproteinase *MMP-23B *were significantly (FDR ≤ 0/05) up-regulated (+1.7 fold) in immune versus naïve sheep on day 5 pi in the microarray analysis (Figure 3e), while *MMP-13 *was down-regulated (-2.3 fold) in the immune group compared to naive at day 5 post-challenge (Figure 3e and Table 5). There was also significant up-regulation of the protease inhibitor cystatin C (*CST3*) in immune sheep compared to naïve at day 0 and day 5 post-challenge (+1.4/+1.5 fold) (Figure 3e and Table 5). The multiplex qRT-PCR analysis confirmed transcripts for MMP-13 were expressed at significantly (*P *≤ 0.05) lower levels in immune versus naïve sheep at day 0, while *MMP23B *gene expression showed a similar pattern at day 2 pi (*P *≤ 0.05; immune vs. naive) (Figure 6). Transcripts for cystatin C appeared to be expressed at high but fairly consistent levels in both groups, although significantly higher levels were detected in naïve sheep than immune sheep at day 2 post-challenge (Figure 6).

### Immunoglobulins

Genes featuring the "immunoglobulin" descriptor occur 31 times among the significantly (FDR ≤ 0.05) up-regulated transcripts in immune animals on day 5 post-challenge compared to day 5 naïve, and 13 times in the day 2 immune versus day 0 immune group (see [34] for complete dataset).

### Gastric lysozyme family

Members of the gastric lysozyme family [38] were the most consistently down-regulated transcripts (FDR ≤ 0.05) detected in the Expt. 1 microarray analyses (day 5 post-challenge) (Figure 3f and Table 5). RT-PCR and sequence analysis have previously indicated infection-associated down-regulation of transcripts for the ovine gastric lysozyme genes *1A, 2A, 3A *and *4A*, and confirmed these transcripts all exhibited some level of polymorphism (Anderson and Knight; data not shown). Gastric lysozymes 1A and 4A were selected for further analysis in the multiplex qRT-PCR assay (Table 3). This analysis confirmed that transcripts for lysozymes 1A and 4A were both highly expressed in the naïve abomasum and were significantly (*P *≤ 0.05-0.005) down-regulated in immune versus naïve sheep at days 2 and 5 post-challenge, although these differences became less apparent from day 10 onwards as levels in the naïve animals reduced (Figure 7).

### MALAT-1

Metastasis-associated lung adenocarcinoma transcript 1 (*MALAT-1*) was identified as among the most significantly (FDR ≤ 0.05) down-regulated transcripts in immune sheep on day 2 post-challenge (-1.8-2 fold), compared to naïve animals or day 0 (Table 4 and Figure 3g). This observation from the array data was confirmed by the multiplex qRT-PCR analysis, with significantly reduced levels of *MALAT-1 *transcripts in immune versus naïve animals at day 0 and day 2 post-challenge (Figure 7). *MALAT-1 *is understood to be a novel non-coding RNA which is highly conserved across species and up-regulated during metastasis, but its function is unknown to date [39]. We also observed transcripts for "boundary element associated factor" as the most highly down-regulated transcript in immune versus naïve animals at day 5 post-challenge (-6.5 fold, Table 5); again the function of this gene product is not clear but it appears to be involved in transcriptional regulation by compartmentalization of the genome [40].

## Discussion

The molecular mechanisms that contribute to protection from gastrointestinal nematodes have been extensively investigated in rodent models (e.g. reviews by [41-43]), but progress in understanding the responses to natural infections in ruminants, especially sheep, has been slower partly due to the limited range of appropriate species-specific reagents and genomics tools. Here we summarise findings using a novel ovine cDNA microarray, which is the first global transcriptomic analysis of ovine immune responses to *Teladorsagia circumcincta *challenge. Our data presented here are consistent with the observed increased adaptive and innate immune response taking place by days 2 and 5 post-challenge in the abomasal mucosa of sheep previously exposed to the parasite using this experimental protocol [11]. As discussed below, many of our findings highlight the importance of genes which have been investigated in rodent models of gastrointestinal nematode infection and previously highlighted in other ruminant studies, such as the expression changes in immunoglobulin transcripts, mucus and mast cell products, and members of the arachidonic acid pathway, and point to common mechanisms operating in response to nematode parasitism of both the gastric and intestinal mucosa. However, we have also highlighted novel changes such as altered expression levels of granulysin, gastric lysozymes, members of the matrix-metalloprotease family, and *STIP1*, which indicate responses that are unique to this model or have not been identified to date.

As reported previously, in all the trials (Table 1), sheep immunised by previous infection ("immune") had significantly (*P *≤ 0.01) lower worm burdens than naïve animals at the same timepoint, even at 48 h post-challenge [4,11]. Similarly, worms recovered from the immunised sheep showed higher levels of stunting [4]. These observations confirmed there were significantly increased anti-parasite responses in the immunised groups compared to naïve, and that these responses manifested themselves within 48 h of challenge. Previous work using materials from these infection trials indicated that there were clear histological and/or histochemical phenotypic differences elicited in the host response between the "naïve" and "immune" groups, such as mucosal hypertrophy, mast cell and eosinophil counts, and in mucous composition ([8,44] and Craig et al., unpublished observations). In order to characterise these differences at a molecular level we examined changes in gene expression at the transcript level in the same experimental animals.

There were some discrepancies in this study between the findings from the cDNA arrays and the multiplex qRT-PCR results, which is likely to be due, in part, to the inability of some of the cDNA probes (+400bp) to distinguish between groups of highly similar genes. This is particularly obvious in the case of the highly similar family of ruminant gastric lysozyme genes [38]; only the specific RT-PCR/competitive qRT-PCR techniques would have been able to reliably distinguish between transcripts for lysozymes *1A *and *4A*, which are 92% identical at the DNA level. A similar explanation is likely to account for inconsistencies between the microarray and qRT-PCR findings in identifying changes in transcripts for members of the C1 complement family, and for the *ITLN *genes *1, 2 *and *3*, which were unlikely to be distinguished by the *ITLN2 *cDNA probe on the array; there were also temporal differences in expression between the three genes (Figure 5). These findings highlight the importance of validation of cDNA array data by more sensitive techniques, such as quantitative PCR-based analyses.

As immunity to *T. circumcincta *is associated with local IgA and IgE responses typical of gastrointestinal nematode infections [4,6] it is unsurprising that genes featuring the "immunoglobulin" descriptor were highly represented among the significantly up-regulated transcripts in immune animals. Our findings for immunoglobulin transcripts are consistent both with an increased adaptive response in sheep previously exposed to the parasite, and with observations from other global analyses of responses to gastrointestinal nematode infection [45-47].

Our findings highlight the universal importance of mucus components in mammalian responses to parasitic nematode infection, such as *CLCA1*, members of the *ITLN *family and trefoil factor 3, which potentially contribute to expulsion by altering mucus composition and making the environment of the parasite inhospitable. A similar pattern of gene expression has been identified in response of the abomasal mucosa to *Haemonchus contortus *infection [46]. There is a clear association between intestinal goblet cell hyperplasia, release of their effector molecules and altered mucus composition with the trapping and expulsion of gastrointestinal nematodes, mostly based on murine studies (reviewed by [43,48,49]. Sheep given trickle infection of *T. circumcincta *are known to exhibit hyperplasia of mucous-neck cells [14], which are phenotypically similar to goblet cells in the intestine, but this occurs as a later event in naïve sheep in response to challenge [10]. Previous work [44] has also demonstrated differences in PAS staining in naïve versus immune sheep (*P *< 0.05, Kruskall-Wallis at day 10 post-challenge) from the experimental samples used in this study, indicating altered abomasal mucous composition between the two groups.

*CLCA1 *(the putative orthologue of *CLCA3 *in mice), which featured as one of the most consistently up-regulated transcripts in immune sheep, is secreted by goblet/mucous-neck cells in association with Th2-type inflammatory responses, such as murine models of pulmonary inflammation and gastro-intestinal nematode infection [50]. We have confirmed that *CLCA1 *is up-regulated by IL-4 and IL-13 in a human goblet cell line [43], and by IL-4 in ovine gastric epithelial cells (Knight et al., in preparation). The significant up-regulation of transcripts for the mucous cell product trefoil factor 3 (*TFF3*) in immune animals compared to naïve was in contrast to *TFF2*, which is highly down-regulated (Tables 4 and 5). *TFF3 *is IL-4/IL-13-regulated and interacts with the mucin MUC2 (not on array) to alter mucus viscosity, with which it co-localises in human intestinal goblet cells, and up-regulation of both these mucus components has been associated with responses to nematode infection in mice (reviewed by [43]). Up-regulation of *TFF3 *and down-regulation of *TFF2 *transcripts has also been demonstrated in the abomasal mucosa of sheep during the response to *H. contortus *[46,51,52], thus switching to a more "intestinal" phenotype in terms of TFF expression.

The multiplex qRT-PCR analyses showed the galactose-binding lectins *ITLN*s 1, 2 and 3 were significantly up-regulated in response to challenge in naïve sheep, with earlier up-regulation of *ITLN3 *than *ITLN*s -1 and -2 (Figure 5). *ITLN *protein is localised to mucous neck cells in the abomasal mucosa, and highly up-regulated in infected compared to worm-free naïve sheep [27,35]. We also observed infection-associated up-regulation of transcripts for two other galactose-binding lectins; galectin 1, which is thought to bind gastrointestinal mucins [53], and galectin 4, which has been shown to be localised to gastric mucous cells in mice [54]. There is a clear temporal association between expression of members of the intelectin family by murine intestinal goblet cells and resistance to gastrointestinal nematode infection in mice [55,56]. The *ITLN *family, like *CLCA1*, are highly regulated by Th2 cytokines in murine and human goblet cells [43], and this has also been confirmed in ovine tracheal goblet cells [57] and gastric epithelial cultures (Knight et al, in preparation). Both *CLCA1 *and intelectin protein were up-regulated in mucosal washings from previously infected, but not naïve, animals [58] indicating they act concordantly as part of an increased Th2-type response to alter mucus composition. It is possible that members of both the intelectin and galectin family are involved in interaction with mucins to change the rheological properties or adhesiveness of the mucus, and/or adhere to the worms to exert effector function or target them for immune clearance.

Significant up-regulation of the high affinity IgE receptor in immune animals day 2 and day 5 post-challenge, along with up-regulation of cathepsin C, is consistent with an increased mast cell response in this group. T-cell mediated mucosal mast cell hyperplasia, accompanied by release of mast-cell mediators, is a characteristic feature of gastrointestinal nematode infections in mammals (reviewed by [43,59]). While numbers of mast cells (globule leucocytes) in the ovine abomasal mucosa are normally very low in uninfected sheep, they markedly increase in response to *T. circumcincta *infection in sensitised sheep, accompanied by release of sheep mast cell protease (SMCP) [13,60]. An assessment of the samples used in this study confirmed that the immune group had significantly higher numbers of mast cells than the naïve group at days 0, 2, 5 and 10 post-challenge, with the naïve animals showing very little increase in mucosal mast cell numbers in response to challenge ([8] and Craig et al., unpublished data).

We see evidence of up-regulation of a number of transcripts associated with eicosanoid metabolism, in particular *PLA2G2A*, which can initiate and regulate inflammation [61]. Increased synthesis of phospholipase and other members of the arachidonic acid cascade have been associated with intestinal nematode infection in rodents, cattle and swine [62-66], and so are likely to play a common key role in the induction and/or regulation of nematode-induced allergic inflammation; we would postulate a similar set of events occurring in the ovine abomasal mucosa in response to parasite challenge. Members of the complement cascade also appear to be up-regulated in response to *T. circumcincta *challenge; up-regulation of the complement C1 and C4 has similarly been linked to resistance to intestinal nematode infection in cattle [65]. Complement activation serves to initiate and propagate pro-inflammatory responses, although the role of complement in immunity to parasitic helminths is unclear [67]. Proteomic analysis has indicated complement and other plasma proteins are up-regulated in the mucosa of *T. circumcinta *infected versus naïve sheep, although these are likely to be derived from plasma rather than local expression [58].

We observed significant up-regulation of the chemokines *CCL2 *and *CCL5 *in response to larval challenge (Table 4, Figures 3c) and 6). *CCL2*, which is produced by a range of cell types including mast cells [68] has been shown to be released locally in response to gastrointestinal nematode infection in mice, and has been implicated in resistance to *Trichuris muris *by steering towards a Th2-type response [69]. *CCL5 *is chemotactic for T cells, eosinophils, and basophils and implicated in a wide range of inflammatory diseases [70]. A previous RT-PCR-based analysis has shown that the abomasal mucosa is a source for a range of chemokine transcripts, and that *CCL5 *transcripts are up-regulated in response to *T. circumcincta *challenge (Griffith et al., unpublished observations).

One of the most surprising findings from this study, which has not been evidenced from rodent models, is the striking up-regulation of transcripts for granulysin in immune animals, which is normally associated with antimicrobial activity [71], along with other transcripts (granzymes, cathepsin C) normally associated with lymphocytes exhibiting cytotoxicity. Granulysin and NK-lysin are secreted, antimicrobial lipid-binding proteins belonging to the saposin family [71]. They have been identified in human, swine and bovine cytotoxic T lymphocytes and natural killer (NK) cells [72,73], but no murine counterpart has been identified to date. Granulysin is active against a broad range of intracellular and extracellular microbes [74], and granulysin activity has also been associated with apoptosis and necrosis of keratinocytes [75]. Granzymes A, B and H, like granulysin, are also normally associated with the activity of cytotoxic T-cells and NK cells [76], while cathepsin C is required for the processing and correct functioning of granzymes A and B and mast cell proteinases [77]. Interestingly, the pattern of expression of granulysin transcripts did not closely parallel that of the granzymes and cathepsin C (Figure 4), which suggests a different cellular source in the abomasal mucosa. This is the first time this molecule has been associated with gastrointestinal nematode infection, and the first association of this molecule with ovine disease. We have recently confirmed increased levels of granulysin protein in the abomasal mucosa of immune, but not naïve, sheep, and the potential biological role of granulysin in nematode infection is under further investigation (Griffith et al, in preparation).

Expression levels of transcripts for a number of heat shock proteins were significantly altered in the microarray analyses; in particular *STIP1*, which facilitates the association of the HSP70/90 complex which is implicated in the folding/regulation of a range of signalling proteins [78], was significantly increased in response to challenge (Figure 6). Host heat shock proteins are expressed constitutively in all cells but synthesis is increased in response to certain stressors or infection [79]. There has been little evidence to identify a direct role for heat shock proteins in gastrointestinal nematode infection, although *T. spiralis *has been shown to elicit host heat shock protein production during muscle migration [80]. The up-regulation and/or release of heat shock proteins is likely to be solely the effect of cellular damage caused to the mucosa by the parasite, but their release may also activate immune cells or facilitate antigen presentation [79,81].

Both the microarray and multiplex qRT-PCR analysis demonstrated significant down-regulation of transcripts for *MMP-13*, as well as apparently high levels of cystatin C transcript expression. In a separate study (Knight et al., unpublished data), we have detected markedly decreased levels of transcripts for *MMP-7 *and *TIMP-1 *(tissue inhibitor of metalloproteinases) in immune versus naïve sheep exposed to *T. circumcincta *(MMP-7 and TIMP-1 were not this on array). Matrix metalloproteinases are implicated in a wide range of processes including regulation of inflammatory responses such as modulating response to cytokines and activation of β-defensins, as well as tissue dissolution/remodelling [82,83] and have been associated with T-cell mediated tissue damage to the gut mucosa [84]. The apparently higher levels of these transcripts in naïve compared to immune sheep may reflect increased tissue damage that is generally observed in the abomasa of animals that have not built up a level of immunity to the parasite. It is also possible that they have a role in inflammatory processes/tissue remodelling that is "replaced" in previously exposed sheep by mast cell proteases, which may have a similar function [85], following expansion of the mucosal mast cell population. The altered profile in matrix metalloproteinases may also have implications in interaction with incoming larvae, as helminth parasites are understood to produce proteinase inhibitors to protect themselves from degradation by host proteinases [86], and parasites of the gastrointestinal tract can interact with host proteolytic pathways with immunomodulatory effects [87,88]. Furthermore, *T. circumcincta *larvae abundantly produce the cysteine protease cathepsin F [89], which raises the possibility that high local levels of cystatin C could serve a protective function. The potential interaction of these matrix-metalloproteases and protease inhibitors with proteases or inhibitors produced by incoming larvae would merit further investigation.

The most consistently down-regulated transcripts in immune versus naïve sheep detected at day 5 post-challenge were members of the ruminant gastric lysozyme family. It should be noted that the array contained more than 50 spots representing members of this family which were down-regulated in the day 5 immune vs. naïve comparison, and 5 spots in the day 5 naïve vs. day 0 naïve comparison. The large numbers of significant spots may be partly due to the overrepresentation of the lysozyme family on the cDNA chip, having a high proportion of sequences in the KN511 library (2.5%) [90]. There are four highly similar ruminant gastric lysozyme genes, which are thought to have evolved from the lysozyme C gene family by gene-duplication events [38]. They are highly expressed in the ovine abomasum and are thought to act as a major digestive enzyme, functional at low pH, for the large amounts of bacteria entering from the rumen, by breaking down peptidoglycan cell walls which cannot be hydrolysed by conventional digestive enzymes. This analysis confirmed that transcripts for lysozymes *1A *and *4A *were both highly expressed in the naïve abomasum and significantly down-regulated in immune versus naïve sheep at days 2 and 5 post-challenge, although these differences became less apparent from day 10 onwards as levels in the naïve animals reduced (Figure 7). This pattern is in contrast to a parallel proteomics study, where lysozyme *4A *increased in previously infected animals compared to naïve at day 0 and day 2 post-challenge (Brown, Pemberton et al., in preparation) and members of the lysozyme family were found to be up-regulated in abomasal epithelial extracts and mucosal washes from previously infected versus naïve sheep [58]. This disparity is likely to be due to differing rates of transcription, processing and storage of the gastric lysozymes prior to secretion. Sheep parasitized by *T. circumcincta *show diminution of parietal cell number, raised abomasal pH and hypergastrinaemia [14,91], so we could speculate that the alterations in lysozyme production, and the possible inability of lysozymes to function in the raised pH of the abomasum, both reflect these changes and may contribute to the resultant nutritional loss seen in infected animals.

In summary, the up-regulated expression of mucous-cell related transcripts such as *CLCA1*, *TFF3 *and *ITLN*s, as well as transcripts for the high affinity IgE receptor, *CCL2*, and members of the arachidonic acid pathway in response to ovine gastric nematode infection, parallel findings from mouse models of intestinal nematode infection by ourselves and others [43,56,92]. Many of these changes reflect a common Th2-driven immune response occurring across species and in both areas of the gastrointestinal tract. However, other findings point to novel changes, such as the distinct local up-regulation of granulysin in immune sheep, a gene which is present in humans, cattle and pigs but not mice, and the alteration in the gastric lysozyme profile which is unique to ruminants.

As can be seen from the pattern of expression of the gastric lysozymes discussed previously, it needs to be borne in mind that changes in the transcriptome may not directly reflect contemporaneous changes in local levels of the corresponding protein, many of which are stored before subsequent release into the local microenvironment. Many of the changes described here are likely to reflect the pronounced cellular changes in the abomasal mucosa induced by exposure to *T. circumcincta *larvae. Nevertheless, the data presented here gives valuable insights into some of the molecular mechanisms that may be operating at the ovine host-parasite interface to control gastric nematode infection.

## Competing interests

The authors declare that they have no competing interests.

## Authors' contributions

PK planned and supervised the study and drafted the manuscript, SG carried out the sequencing and RT-PCR analysis of granulysin, JP assisted with post-mortem sample collection and carried out RNA extractions/QC checks, AP assisted with interpretation of data and participated in manuscript preparation, LG carried out the sequence analysis of MMPs and inhibitors, KA carried out the sequence analysis of the lysozyme family, RT supervised the microarray experiments, SS carried out the hybridisations, DW carried out statistical analysis of the microarray experiments, MF carried out annotation of the ARK-Genomics *O. aries *12K v1.0 array and assisted with data processing, AA supervised the microarray experiments and advised on manuscript preparation, SB carried out the Ingenuity analysis and assisted with presentation of the output, DS supervised the experimental infections and advised on manuscript preparation, HM and IM conceived and obtained the funding for the VTRI overall project and HM advised on manuscript preparation. All authors read and approved the final manuscript.

## Supplementary Material

Additional file 1 **Table S1 Details of primers and PCR conditions used for RT-PCR analysis**. Details of primers and PCR conditions used for RT-PCR analysis, and sequences used to design PCR probes. **Table S2** Total numbers of genes whose expression levels were significantly altered detected in each hybridisation experiment Total numbers of genes whose expression levels were significantly altered (Benjamini & Hochberg FDR ≤0.05) detected in each hybridisation experiment; (Table S2a: Expt.1; day 5 post-challenge and Table S2b: Expt. 2; day 2 post-challenge) including dye interactions. Click here for file

Additional file 2 **Table S3 Significantly highly represented networks identified using Ingenuity Pathways Analysis software (Ingenuity^® ^Systems**, [26]**)**. Networks that were significantly highly represented (P≤10-10; Fischer's exact test) identified from the Imd0/Nvd0 and Imd2/Imd0 datasets from Expt. 2 (see Table II), using Ingenuity Pathways Analysis software (Ingenuity^® ^Systems, [26]). **Table S4** Canonical pathways identified using Ingenuity Pathways Analysis software (Ingenuity^® ^Systems, http://www.ingenuity.com). The top 20 most significant canonical pathways from the Imd0/Nvd0 and Imd2/Imd0 datasets from Expt. 2 (See Table II), identified using Ingenuity Pathways Analysis software (Ingenuity^® ^Systems, [26]), are shown. Click here for file
