# Supplementary material for: Novel gene expression responses in the ovine abomasal mucosa to infection with the gastric nematode Teladorsagia circumcincta
Source: Vet Res. 2011 Jun 17;42(1):78. doi: 10.1186/1297-9716-42-78 (PMC3135528; doi:10.1186/1297-9716-42-78)
Supplement: Additional file 1 — Table S1 Details of primers and PCR conditions used for RT-PCR analysis. Details of primers and PCR conditions used for RT-PCR analysis, and sequences used to design PCR probes. Table S2 Total numbers of genes whose expression levels were significantly altered detected in each hybridisation experiment Total numbers of genes whose expression levels were significantly altered (Benjamini & Hochberg FDR ≤0.05) detected in each hybridisation experiment; (Table S2a: Expt.1; day 5 post-challenge and Table S2b: Expt. 2; day 2 post-challenge) including dye interactions. [file 1297-9716-42-78-S1.DOC]

**Table S1: Details of primers and PCR conditions used for RT-PCR analysis.**

Details of primers and PCR conditions used for RT-PCR analysis, and sequences used to design PCR probes. Cycling conditions for each RT-PCR were 2 minutes at 94^o^C, followed by 40 s at 94^o^C, 40s at annealing temperature (^a^) and120s at 72^o^C for required number of cycles (^b^), followed by 7 mins at 72^o^C.

| Functional Category | Gene Name | EMBL acc. No. | Primers | PCR conditions | |
| --- | --- | --- | --- | --- | --- |
|  |  |  |  | Annealing temp ^o^C^a^ | No.  cycles^b^ |
| Cytotoxicity | GNLY | FE028690 | 5’- CCTTTTCCGGTCTGGCTCCT  3’- CGGACGTTTCAGCAGCTTCA | 60 | 22 |
|  | CatC | DY479012 | 5’- TGGGGACGTGTGCAGTATTCC  3’- CACTGAGTGGCCCTATGTGGTC | 58 | 22 |
|  | GZMA | FE037399 | 5’- CTTGCAACGCTCTGAACTTCACATA  3’- TCTCGGGACGTCTCTCACCA | 60 | 32 |
|  | GZMB | FE021442 | 5’- TCAGGACGTCCGGCATAGGT  3’- CTGGGTGGCAGATGGCTCTT | 60 | 32 |
|  | GZMH | FE026184 | 5’- ATCAGGGTGACTCCGGTGGA  3’- GCCCCAGGACAGTGGTCAGT | 60 | 32 |
| Mucus composition | CLCA1 | EE748540 | 5’- TCCCAAAGGAGGCCAACTCA  3’- CAGGAAAACTCAGCCCAAGGAA | 58 | 28 |
|  | ITLN1 | AM087961 | 5’- GCGAGAATTTGTTGCAGGAT  3’- TGGCTTTCTGTCTCACACCA | 58 | 25 |
|  | ITLN2 | EF521881 | 5’- GCGAGAATTTGTTGCAGGAT  3’- TGGGTTCACATCTCATAGA | 58 | 23 |
|  | ITLN3 | AM888394 | 5’- GCGAGAATTTGTTGCAGGAT  3’- TGGGTTCCACATCTCATAGA | 58 | 25 |
| Heat shock response | HSPA8 | DY491193 | 5’- GGAGCTCCTCCATCAGGTGGT  3’- TCCATGTGCACACACATTCCA | 58 | 22 |
|  | HSPCA | FE020983 | 5’- GGAGGATCCCCAGACACACG  3’- TTGGAGGGAACGGAGACAGC | 58 | 22 |
|  | ST1 | FE037366 | 5’- CAAGAGCGGCTGGCCTACAT  3’- TGCACTCCTCGCAGTCCTTG | 58 | 28 |
| Pro-inflammatory response | PLA2G2A | FE034658 | 5’- GCGGCAATGATCAAGCACAC  3’- CGGCGGATGGACACATTGTA | 58 | 22 |
|  | CF1 | FE024140 | 5’- CTGCCGCACATTGTGTCAGA  3’- GACACAGGCAGGGACGGAGT | 58 | 31 |
|  | CCL2 | FE031007 | 5’- CCAATCCAGAGGCCAACAGC  3’- TCAGGTTGGGGTCTGCACAA | 58 | 31 |
| Tissue remodelling  Digestion | MMP13 | AY091604 | 5’- CGCTAGCAGTGAGGACGGAAG  3’- TCCAAGGTAAACTGGACAATTAAAACA | 58 | 28 |
|  | MMP23 | DY483383 | 5’- CCACACGGACTGCCTGGTCT  3’- TCTGACCGCAACGGAAGGTC | 60 | 34 |
|  | CST3 | FE030675 | 5’- ATGACCAGCCGCACCTGAAG  3’- CACCGTGCACTTGGAAAGCA | 58 | 24 |
|  | LZM1A | M32492 | 5’- ggcaaaacccctaacgcagt  3’- acttatctgcctctgaatttggtca | 58 | 22 |
|  | LZM4A | M32497 | 5’-tcggactggatgactataaggga  3’-ccttcatgatcagcaggatgc | 55 | 24 |
| Unknown  “Housekeeping” genes | MALAT | FE036272 | 5’- GCCGTCTGCCAATCTTTCGT  3’- GCCGTCTGCCAATCTTTCGT | 58 | 24 |
|  | ATPase | X02813 | 5’- GCTGACTTGGTCATCTGC  3’- CAGGTAGGTTTGAGGGGATAC | 60 | 31 |
|  | RW1 | FE026635 | 5’- CCACTCCCCACCCTCCTCAT  3’- TGCAGATGGTCTCAGCCGAAT | 58 | 30 |
|  | TM57 | FE030125 | 5’- GCTGAAAGTTCGGGAGCTTCG  3’- GTCCAGAGGGGCTGGTCTCC | 58 | 30 |

**Table S2: Total numbers of genes whose expression levels were significantly altered detected in each hybridisation experiment.**

Total numbers of genes whose expression levels were significantly altered (Benjamini & Hochberg FDR≤ 0.05) detected in each hybridisation experiment; (Table S2a: Expt.1; day 5 post-challenge and Table S2b: Expt. 2; day 2 post-challenge) including dye interactions. Nv = “naïve” yearlings, worm naïve prior to challenge; Im = “immune” yearlings, previously infected by trickle infection prior to challenge; d0, d2, d5= days post-challenge. The comparisons refer to the hybridisation designs listed in Tables II of the manuscript; R_G refers to total numbers of genes whose expression levels were significantly altered as a result of treatment-by-dye interactions. Any transcripts showing significant (FDR≤ 0.05) treatment-by-dye interactions were subsequently removed from the gene lists. This precautionary measure excluded pairs of treatment estimates (from cy5-cy3 or cy3-cy5 dye orientations) with conflicting signs and some pairs of treatment estimates with the largest proportionate differences in magnitude. The complete gene lists of significantly differentially expressed genes, with significant treatment-by-dye interactions excluded, are available under Array Express [34] or by contacting the authors.

**Table S2a; Expt. 1 (Day 5 post-challenge)**

| **Comparison** | **Up-regulated** | **Not changed** | **Down-regulated** | **TOTAL** |
| --- | --- | --- | --- | --- |
| Nvd5/Nvd0 | 332 | 9528 | 365 | 697 |
| Imd5/Nvd5 | 965 | 8158 | 1102 | 2067 |
| Nvd5/Nvd0_R_G | 1 | 10224 | 0 | 1 |
| Imd5/Nvd5_R_G | 1 | 10224 | 0 | 1 |

**Table S2b; Expt 2 (Day 2 post-challenge)**

| **Comparison** | **Up-regulated** | **Not changed** | **Down-regulated** | **TOTAL** |
| --- | --- | --- | --- | --- |
| Imd2/Imd0 | 1090 | 7948 | 1187 | 2277 |
| Nvd2/Nvd0 | 0 | 10225 | 0 | 0 |
| Imd0/Nvd0 | 66 | 10115 | 44 | 110 |
| (Imd2/Imd0)/(Nvd2/Nvd0) | 486 | 9084 | 655 | 1141 |
| Imd2/Imd0_G_R | 553 | 9209 | 463 | 1016 |
| Nvd2/Nvd0_G_R | 42 | 10128 | 55 | 97 |
| Imd0/Nvd0_G_R | 541 | 9173 | 511 | 1052 |
| (Imd2/Imd0)/(Nvd2/Nvd0)_G_R | 0 | 10225 | 0 | 0 |
