# Supplementary material for: Novel gene expression responses in the ovine abomasal mucosa to infection with the gastric nematode Teladorsagia circumcincta
Source: Vet Res. 2011 Jun 17;42(1):78. doi: 10.1186/1297-9716-42-78 (PMC3135528; doi:10.1186/1297-9716-42-78)
Supplement: Additional file 2 — Table S3 Significantly highly represented networks identified using Ingenuity Pathways Analysis software (Ingenuity® Systems, [26]). Networks that were significantly highly represented (P≤10-10; Fischer's exact test) identified from the Imd0/Nvd0 and Imd2/Imd0 datasets from Expt. 2 (see Table II), using Ingenuity Pathways Analysis software (Ingenuity® Systems, [26]). Table S4 Canonical pathways identified using Ingenuity Pathways Analysis software (Ingenuity® Systems, http://www.ingenuity.com). The top 20 most significant canonical pathways from the Imd0/Nvd0 and Imd2/Imd0 datasets from Expt. 2 (See Table II), identified using Ingenuity Pathways Analysis software (Ingenuity® Systems, [26]), are shown. [file 1297-9716-42-78-S2.DOC]

**Table S3: Significantly highly represented networks identified using Ingenuity Pathways Analysis software**

**(Ingenuity® Systems [26]).** Networks that were significantly highly represented (P≤10^-10^; Fischer’s exact test) identified from the

Imd0/Nvd0 and Imd2/Imd0 datasets from Expt. 2 (see Table 2 of manuscript), using Ingenuity Pathways Analysis software

(Ingenuity® Systems [26]). Focus molecules are the numbers of molecules from each dataset that are present in the

network, while the network score describes the probability (P=10^-network score^) that the molecules in the network are associated with the

dataset by chance alone. Networks with a score of +30 were viewed as highly significant (10 = minimum score).

**Table S4: Canonical pathways identified using Ingenuity Pathways Analysis software (Ingenuity® Systems, [26]).** The top 20 most significant canonical pathways from the Imd0/Nvd0 and Imd2/Imd0 datasets from Expt. 2 (see Table 2 of manuscript), identified using Ingenuity Pathways Analysis software (Ingenuity® Systems [26]), are shown. Pathways were identified from the Ingenuity Pathways Analysis library of canonical pathways that were most significant to the data set. The significance of the association between the data set and the canonical pathway was measured in two ways as shown: 1) -log(p-value); Fisher’s exact test was used to calculate a p-value determining the probability that the association between the genes in the dataset and the canonical pathway is explained by chance alone. 2) Ratio- this is the ratio of the number of molecules from the data set that map to the pathway divided by the total number of molecules that map to the canonical pathway is displayed. Molecule- this is the list of molecules in the dataset that appear in the pathway. © 2000-2010 Ingenuity Systems, Inc.
